# Supplementary material for: Reliable protein-protein docking with AlphaFold, Rosetta, and replica-exchange
Source: bioRxiv. 2023 Nov 25:2023.07.28.551063. Originally published 2023 Jul 29. Preprint. [Version 2] doi: 10.1101/2023.07.28.551063 (PMC10402144; doi:10.1101/2023.07.28.551063)
Supplement: Supplementary file 1 [file media-1.pdf]

Supplementary Information Appendix

From sequences to structure to complexes: an *in-silico* pipeline for protein-protein docking

Ameya Harmalkar[a], **Sergey Lyskov**[a], **Jeffrey J. Gray**[a,b1]

[a]Department of Chemical and Biomolecular Engineering, The Johns Hopkins University, Baltimore, MD 21218, USA.; [b]Program in Molecular Biophysics, The Johns Hopkins University, Baltimore, MD 21218, USA.

[2]To whom correspondence should be addressed. E-mail: jgray@jhu.edu

Table S1
SI References

Supplementary Methods

Benchmark Sets.

The benchmark set was curated from Docking Benchmark 5.5((1)), with targets classified based on their extent of flexibility, i.e., rigid, medium, and difficult. We also curated a subset of only antigen-antibody/nanobody targets from the overall set. For each target in the benchmark set, a FASTA sequence was obtained with individual chains separated by a colon (:) indicating chain break. This sequence was used for AlphaFold-multimer (AFm) structure prediction. For target 1N2C, AFm could not generate a structural prediction owing to the longer sequence length and is excluded from the benchmark. For each structural prediction that was generated, comparisons were made to its corresponding bound and unbound forms as obtained from the Docking Benchmark 5.5.

Metrics and evaluation.

The docking performance was evaluated based on interface RMSD (I-rms), fraction of native-like contacts ($f_{\texttt{nat}}$), CAPRI quality, and DockQ scores. These metrics are defined as follows:

Interface RMSD (I-rms): The root-mean-sqaure-deviation (RMSD) of all atoms on the interface in a docked protein structure relative to a reference structure (native). Interface residues are defined as all amino acid residues within 10 Å of any residue on the binding partner.

fraction of native-like contacts ($f_{\texttt{nat}}$): The fraction of native-like contacts recovered in the docked structure relative to the reference structure (native).

CAPRI quality: A CAPRI-based rank calculated on the basis of I-rms, $f_{\texttt{nat}}$, and ligand-RMSD to classify a docked model as incorrect, acceptable, medium, or high-quality prediction.

DockQ Score: Similar to CAPRI-quality, the DockQ scores estimates a score ($\in [0, 1]$) estimating the accuracy of the docked complexes. We calculated this score based on the methodology described in Basu *et al.*(2)

Interface score (Isc): The interface score is analogous to thermodynamic binding energy of protein association. This score is estimated by calculating the total score (gibbs free energy) of protein complex and then by subtracting individual (monomeric) scores of protein partners in absence of its partner. Mathematically, for proteins A and B forming a complex AB, it can be defined as follows:

$$\Delta\Delta G_{\text{interface}} = \Delta G_{\text{AB}} - \Delta G_{\text{A}} - \Delta G_{\text{B}}$$

Supplementary Results

Data availability.

AlphaRED utilizes ColabFold for structure prediction with Rosetta-based docking. The source code for AlphaRED is available on github (github.com/Graylab/AlphaRED). To ensure ease of availabilty for researchers, we have implemented an online server on the Gray Lab ROSIE server (rosie.graylab.jhu.edu). The server would implement the AlphaRED pipeline and for input sequences would provide docked models with Rosetta energies for further analysis. We expect this implementation to be a great resource for modeling and better understanding protein-protein interactions.

Table S1. Performance of AlphaRED and AFm on Docking Benchmark Set 5.5

PDB	Interface RMSD		f_{nat}		CAPRI Rank		Flexibility
	AF2	AlphaRED	AF2	AlphaRED	AF2	AlphaRED	
1ACB	2.51	2.02	0.86	0.85	1	1	Difficult
1ATN	1.35	1.29	0.87	0.89	2	2	Difficult
1BKD	1.64	1.48	0.84	0.83	2	2	Difficult
1DE4	4.43	5.91	0.37	0.12	0	0	Difficult
1E4K	16.83	5.11	0.03	0.09	0	0	Difficult
1EER	1.75	1.64	0.80	0.81	2	2	Difficult
1F6M	2.76	2.75	0.50	0.56	1	1	Difficult
1FAK	1.71	1.91	0.71	0.71	2	2	Difficult
1FQ1	4.06	3.24	0.50	0.56	0	1	Difficult
1H1V	3.61	3.63	0.22	0.22	1	1	Difficult
1IBR	1.34	1.31	0.79	0.89	2	2	Difficult
1IRA	2.76	2.42	0.52	0.62	1	1	Difficult
1JK9	1.70	1.64	0.79	0.87	2	2	Difficult
1JMO	4.10	3.69	0.72	0.82	0	1	Difficult
1JZD	17.62	17.60	0.00	0.00	0	0	Difficult
1PXV	1.30	1.37	0.89	0.91	2	2	Difficult
1R8S	2.12	2.06	0.74	0.75	1	1	Difficult
1RKE	4.31	4.12	0.57	0.64	0	0	Difficult
1Y64	7.44	6.93	0.40	0.35	0	0	Difficult
1ZLI	1.84	1.79	0.86	0.88	2	2	Difficult
2C0L	1.36	1.21	0.89	0.90	2	2	Difficult
2FJG	17.03	5.65	0.02	0.07	0	0	Difficult
2I9B	3.98	3.03	0.59	0.66	1	1	Difficult
2J7P	2.74	2.76	0.38	0.43	1	1	Difficult
2O3B	1.27	1.29	0.73	0.81	2	2	Difficult
2O3B	1.16	1.16	0.81	0.85	2	2	Difficult
2OT3	1.97	2.01	0.69	0.70	2	1	Difficult
3AAD	22.37	21.03	0.00	0.00	0	0	Difficult
3AAD	21.60	21.48	0.00	0.00	0	0	Difficult
3F1P	16.89	12.14	0.00	0.00	0	0	Difficult
3FN1	1.93	1.94	0.75	0.77	2	2	Difficult
3H11	5.89	6.00	0.35	0.55	0	0	Difficult
4DW2	18.37	7.00	0.00	0.02	0	0	Difficult
4GAM	28.34	28.11	0.00	0.00	0	0	Difficult
5C7X	3.76	2.80	0.23	0.48	1	1	Difficult
1B6C	5.30	4.24	0.39	0.31	0	0	Medium
1CGI	1.66	1.63	0.86	0.86	2	2	Medium

PDB	Interface RMSD		f_{nat}		CAPRI Rank		Flexibility
	AF2	AlphaRED	AF2	AlphaRED	AF2	AlphaRED	
**1FC2**	1.96	1.87	0.84	0.88	2	2	Medium
**1GP2**	2.33	1.93	0.48	0.55	1	2	Medium
**1GRN**	1.80	1.72	0.78	0.76	2	2	Medium
**1HE8**	3.48	3.42	0.77	0.77	1	1	Medium
**1I2M**	3.03	3.10	0.74	0.66	1	1	Medium
**1IB1**	17.08	5.07	0.00	0.22	0	0	Medium
**1IJK**	5.18	5.23	0.78	0.72	0	0	Medium
**1JIW**	1.53	1.41	0.63	0.75	2	2	Medium
**1K5D**	1.55	1.57	0.75	0.75	2	2	Medium
**1KKL**	20.46	17.77	0.02	0.00	0	0	Medium
**1LFD**	1.00	1.07	0.88	0.94	2	2	Medium
**1M10**	4.40	5.40	0.27	0.33	0	0	Medium
**1MQ8**	1.97	1.58	0.73	0.94	2	2	Medium
**1NW9**	13.89	4.93	0.00	0.34	0	0	Medium
**1R6Q**	1.06	1.02	0.94	0.89	2	2	Medium
**1SYX**	1.22	1.17	0.79	0.90	2	2	Medium
**1WQ1**	1.54	1.45	0.68	0.79	2	2	Medium
**1XQS**	1.91	1.71	0.75	0.87	2	2	Medium
**1ZM4**	2.59	2.56	0.63	0.68	1	1	Medium
**2CFH**	3.09	2.89	0.75	0.63	0	1	Medium
**2DD8**	13.56	1.70	0.00	0.78	0	2	Medium
**2H7V**	2.26	2.55	0.68	0.73	1	1	Medium
**2HRK**	0.99	0.96	0.94	0.91	3	3	Medium
**2NZ8**	1.14	1.02	0.86	0.92	2	2	Medium
**2OZA**	13.24	8.20	0.00	0.17	0	0	Medium
**2Z0E**	1.25	1.37	0.93	0.93	2	2	Medium
**3AAA**	1.33	1.32	0.77	0.94	2	2	Medium
**3AAD**	22.37	15.42	0.00	0.00	0	0	Medium
**3BX7**	2.11	2.08	0.63	0.65	1	1	Medium
**3CPH**	5.63	4.09	0.14	0.21	0	0	Medium
**3DAW**	1.31	1.26	0.84	0.84	2	2	Medium
**3EO1**	21.58	3.63	0.00	0.33	0	1	Medium
**3G6D**	15.14	13.94	0.00	0.00	0	0	Medium
**3HI6**	4.89	4.52	0.17	0.26	0	0	Medium
**3L5W**	14.90	11.63	0.00	0.00	0	0	Medium
**3R9A**	19.05	4.63	0.00	0.18	0	0	Medium
**3RJQ**	9.77	4.75	0.00	0.21	0	0	Medium

PDB	Interface RMSD		f_{nat}		CAPRI Rank		Flexibility
	AF2	AlphaRED	AF2	AlphaRED	AF2	AlphaRED	
**3S9D**	1.73	1.36	0.42	0.79	2	2	Medium
**3SZK**	14.92	7.40	0.00	0.00	0	0	Medium
**3V6Z**	17.17	6.25	0.00	0.06	0	0	Medium
**4ETQ**	20.17	5.91	0.00	0.15	0	0	Medium
**4FZA**	2.36	2.08	0.80	0.85	1	1	Medium
**4IZ7**	1.88	1.94	0.72	0.75	2	2	Medium
**4JCV**	20.06	11.80	0.00	0.02	0	0	Medium
**4LW4**	3.03	3.63	0.67	0.33	1	1	Medium
**4M3K**	16.13	2.06	0.00	0.46	0	1	Medium
**4POU**	13.79	2.43	0.00	0.51	0	1	Medium
**5CBA**	2.43	2.42	0.82	0.82	1	1	Medium
**5E5M**	5.26	3.15	0.04	0.40	0	1	Medium
**5HGG**	4.85	3.38	0.25	0.21	0	1	Medium
**5HYS**	16.42	9.16	0.00	0.00	0	0	Medium
**5KOV**	16.97	7.21	0.00	0.19	0	0	Medium
**5VNW**	29.75	2.58	0.00	0.42	0	1	Medium
**5WHK**	2.66	2.55	0.57	0.67	0	1	Medium
**6A0Z**	13.99	4.87	0.10	0.18	0	0	Medium
**6AL0**	3.09	2.78	0.44	0.52	0	1	Medium
**6EY6**	14.81	12.61	0.14	0.15	0	0	Medium
**1A2K**	24.77	5.79	0.00	0.14	0	0	Rigid
**1AHW**	1.93	1.41	0.54	0.79	2	2	Rigid
**1AK4**	7.37	7.37	0.17	0.23	0	0	Rigid
**1AKJ**	1.65	1.78	0.75	0.72	2	2	Rigid
**1AVX**	1.42	1.65	0.94	0.92	2	2	Rigid
**1AY7**	0.78	0.77	0.91	0.91	3	3	Rigid
**1AZS**	2.90	2.80	0.66	0.66	1	1	Rigid
**1BUH**	1.38	1.30	0.77	0.74	2	2	Rigid
**1BVN**	1.44	1.13	0.65	0.86	2	2	Rigid
**1CLV**	8.57	4.95	0.07	0.20	0	0	Rigid
**1D6R**	1.07	1.09	0.90	0.84	2	2	Rigid
**1DFJ**	1.45	1.57	0.77	0.83	2	2	Rigid
**1DQJ**	7.43	5.15	0.03	0.15	0	0	Rigid
**1E6E**	1.97	1.89	0.85	0.80	2	2	Rigid
**1E6J**	14.57	12.09	0.00	0.03	0	0	Rigid
**1E96**	1.49	1.23	0.86	0.92	2	2	Rigid
**1EAW**	1.11	1.19	0.78	0.81	2	2	Rigid

PDB	Interface RMSD		f_{nat}		CAPRI Rank		Flexibility
	AF2	AlphaRED	AF2	AlphaRED	AF2	AlphaRED	
**1EFN**	15.08	3.34	0.00	0.33	0	1	Rigid
**1EWY**	5.70	4.60	0.06	0.11	0	0	Rigid
**1EXB**	23.79	19.64	0.00	0.05	0	0	Rigid
**1EZU**	25.50	12.81	0.00	0.01	0	0	Rigid
**1F34**	1.94	1.94	0.73	0.71	2	2	Rigid
**1F51**	19.73	19.91	0.00	0.00	0	0	Rigid
**1FCC**	27.07	1.76	0.00	0.74	0	2	Rigid
**1FFW**	1.41	1.28	0.86	0.96	2	2	Rigid
**1FLE**	1.52	1.46	0.81	0.91	2	2	Rigid
**1FQJ**	2.20	2.30	0.74	0.76	1	1	Rigid
**1GCQ**	14.19	4.55	0.00	0.15	0	0	Rigid
**1GHQ**	13.10	12.54	0.00	0.00	0	0	Rigid
**1GL1**	0.80	0.94	0.90	0.87	3	3	Rigid
**1GLA**	4.12	3.10	0.22	0.30	0	1	Rigid
**1GPW**	1.45	1.38	0.89	0.87	2	2	Rigid
**1GXD**	25.92	3.05	0.00	0.51	0	1	Rigid
**1H9D**	1.57	1.46	0.84	0.89	2	2	Rigid
**1HCF**	14.46	14.56	0.00	0.00	0	0	Rigid
**1HE1**	1.80	1.73	0.78	0.82	2	2	Rigid
**1HIA**	1.19	1.23	0.87	0.95	2	2	Rigid
**1I4D**	20.11	6.39	0.00	0.16	0	0	Rigid
**1J2J**	1.21	1.18	0.88	0.92	2	2	Rigid
**1JPS**	16.85	11.19	0.02	0.04	0	0	Rigid
**1JTD**	1.15	0.94	0.75	0.83	2	3	Rigid
**1JTG**	2.29	2.10	0.60	0.60	1	1	Rigid
**1JWH**	1.78	1.63	0.86	0.83	2	2	Rigid
**1K74**	1.75	1.78	0.87	0.87	2	2	Rigid
**1KAC**	18.09	3.05	0.00	0.34	0	1	Rigid
**1KLU**	13.54	3.04	0.00	0.56	0	1	Rigid
**1KTZ**	1.06	1.06	0.89	0.93	2	2	Rigid
**1KXP**	1.17	1.17	0.85	0.83	2	2	Rigid
**1M27**	8.25	1.90	0.15	0.90	0	2	Rigid
**1MAH**	1.42	1.42	0.78	0.79	2	2	Rigid
**1ML0**	19.64	17.41	0.00	0.02	0	0	Rigid
**1MLC**	10.89	3.99	0.00	0.15	0	1	Rigid
**1OC0**	1.11	1.31	0.94	0.85	2	2	Rigid
**1OFU**	1.34	1.28	0.94	0.94	0	2	Rigid

PDB	Interface RMSD		f_{nat}		CAPRI Rank		Flexibility
	AF2	AlphaRED	AF2	AlphaRED	AF2	AlphaRED	
**1OPH**	2.11	1.48	0.82	0.86	1	2	Rigid
**1OYV**	8.29	6.34	0.68	0.78	0	0	Rigid
**1PPE**	2.00	1.88	0.82	0.82	2	2	Rigid
**1PVH**	18.12	2.88	0.00	0.35	0	1	Rigid
**1QA9**	1.76	1.63	0.64	0.77	2	2	Rigid
**1R0R**	0.86	0.89	0.91	0.93	3	3	Rigid
**1RLB**	19.55	19.32	0.00	0.00	0	0	Rigid
**1RV6**	23.47	22.98	0.00	0.00	0	0	Rigid
**1S1Q**	14.25	2.42	0.00	0.49	0	1	Rigid
**1S78**	27.14	10.50	0.00	0.06	0	0	Rigid
**1SBB**	25.69	3.49	0.00	0.41	0	1	Rigid
**1T6B**	12.23	7.85	0.02	0.20	0	0	Rigid
**1TMQ**	12.66	12.73	0.05	0.08	0	0	Rigid
**1UDI**	1.23	1.16	0.77	0.92	2	2	Rigid
**1US7**	0.76	0.76	0.93	0.94	0	3	Rigid
**1VFB**	13.92	2.07	0.00	0.61	0	1	Rigid
**1WDW**	35.72	35.36	0.00	0.00	0	0	Rigid
**1WEJ**	16.95	9.41	0.00	0.07	0	0	Rigid
**1XD3**	1.72	1.71	0.94	0.89	2	2	Rigid
**1XU1**	13.59	13.07	0.00	0.00	0	0	Rigid
**1YVB**	1.14	1.12	0.83	0.87	2	2	Rigid
**1Z0K**	1.75	1.53	0.65	0.81	2	2	Rigid
**1Z5Y**	2.12	2.12	0.81	0.86	1	1	Rigid
**1ZHH**	16.31	16.53	0.00	0.00	0	0	Rigid
**1ZHI**	1.06	1.22	0.94	0.91	2	2	Rigid
**2A1A**	1.88	1.95	0.84	0.75	2	2	Rigid
**2A5T**	1.48	1.42	0.83	0.85	2	2	Rigid
**2A9K**	16.93	4.29	0.00	0.31	0	0	Rigid
**2ABZ**	8.93	6.97	0.08	0.10	0	0	Rigid
**2AJF**	16.70	5.00	0.00	0.22	0	0	Rigid
**2AYO**	2.10	2.11	0.68	0.57	1	1	Rigid
**2B42**	1.22	1.21	0.82	0.87	2	2	Rigid
**2B4J**	18.53	3.72	0.00	0.26	0	1	Rigid
**2BTF**	1.15	1.11	0.82	0.85	2	2	Rigid
**2FD6**	16.35	12.52	0.00	0.03	0	0	Rigid
**2FJU**	31.69	2.15	0.00	0.59	0	1	Rigid
**2G77**	1.87	2.16	0.75	0.52	2	1	Rigid

PDB	Interface RMSD		f_{nat}		CAPRI Rank		Flexibility
	AF2	AlphaRED	AF2	AlphaRED	AF2	AlphaRED	
**2GAF**	2.45	2.94	0.53	0.44	1	1	Rigid
**2GTP**	1.00	0.96	0.79	0.90	3	3	Rigid
**2HLE**	2.18	1.92	0.64	0.79	1	2	Rigid
**2HQS**	2.49	2.38	0.56	0.60	1	1	Rigid
**2I25**	7.45	2.70	0.11	0.36	0	1	Rigid
**2J0T**	1.15	1.16	0.95	0.95	2	2	Rigid
**2MTA**	22.03	20.25	0.00	0.00	0	0	Rigid
**2O8V**	7.93	7.29	0.20	0.40	0	0	Rigid
**2OOB**	9.07	2.22	0.00	0.55	0	1	Rigid
**2OOR**	19.99	19.61	0.00	0.00	0	0	Rigid
**2OUL**	0.86	0.85	0.94	0.93	3	3	Rigid
**2PCC**	1.28	1.47	0.90	0.85	2	2	Rigid
**2SIC**	0.94	0.89	0.88	0.91	3	3	Rigid
**2SNI**	1.03	0.99	0.84	0.89	2	3	Rigid
**2UUY**	1.01	0.96	0.90	0.94	2	3	Rigid
**2VDB**	0.94	0.86	0.83	0.89	3	3	Rigid
**2VIS**	12.94	7.07	0.00	0.02	0	0	Rigid
**2VXT**	2.20	2.14	0.82	0.86	1	1	Rigid
**2W9E**	13.91	2.27	0.00	0.62	0	1	Rigid
**2X9A**	0.98	0.94	0.89	0.89	3	3	Rigid
**2YVJ**	3.74	1.65	0.10	0.45	1	2	Rigid
**3A4S**	1.64	1.68	0.85	0.90	2	2	Rigid
**3BIW**	11.77	10.32	0.10	0.10	0	0	Rigid
**3BP8**	32.31	4.38	0.00	0.44	0	0	Rigid
**3D5S**	1.85	1.67	0.73	0.76	2	2	Rigid
**3EOA**	17.43	11.54	0.00	0.09	0	0	Rigid
**3H2V**	6.98	2.07	0.00	0.63	0	1	Rigid
**3HMX**	19.54	11.61	0.00	0.03	0	0	Rigid
**3K75**	1.20	1.12	0.89	0.92	2	2	Rigid
**3LVK**	2.26	1.76	0.55	0.73	1	2	Rigid
**3MJ9**	8.22	5.11	0.02	0.13	0	0	Rigid
**3MXW**	12.45	11.22	0.00	0.00	0	0	Rigid
**3P57**	12.61	1.85	0.00	0.96	0	2	Rigid
**3PC8**	1.41	1.32	1.00	1.00	2	2	Rigid
**3RVW**	11.04	5.64	0.00	0.14	0	0	Rigid
**3SE8**	2.58	2.61	0.62	0.62	1	1	Rigid
**3SGQ**	1.56	1.54	0.83	0.83	2	2	Rigid

PDB	Interface RMSD		f_{nat}		CAPRI Rank		Flexibility
	AF2	AlphaRED	AF2	AlphaRED	AF2	AlphaRED	
**3U7Y**	2.17	2.07	0.86	0.89	1	1	Rigid
**3VLB**	1.13	0.98	0.87	0.90	2	3	Rigid
**3WD5**	11.37	11.59	0.00	0.00	0	0	Rigid
**4CPA**	2.07	1.96	0.56	0.69	1	2	Rigid
**4DN4**	12.22	12.14	0.00	0.00	0	0	Rigid
**4FP8**	15.80	5.92	0.00	0.17	0	0	Rigid
**4FQI**	18.79	18.38	0.00	0.00	0	0	Rigid
**4G6J**	11.68	3.36	0.00	0.25	0	1	Rigid
**4G6M**	7.13	3.01	0.03	0.27	0	1	Rigid
**4GXU**	18.48	18.34	0.00	0.00	0	0	Rigid
**4H03**	2.13	2.01	0.43	0.64	1	1	Rigid
**4HX3**	15.67	15.51	0.00	0.00	0	0	Rigid
**4M5Z**	1.16	1.40	0.88	0.84	2	2	Rigid
**4M76**	19.94	4.24	0.00	0.15	0	0	Rigid
**4Y7M**	4.94	3.80	0.24	0.34	0	1	Rigid
**5GRJ**	13.59	3.60	0.04	0.35	0	1	Rigid
**5JMO**	15.48	5.05	0.00	0.14	0	0	Rigid
**5O14**	18.13	3.80	0.00	0.17	0	1	Rigid
**5O1R**	1.24	1.18	0.86	0.90	2	2	Rigid
**5SV3**	1.29	1.26	0.89	0.92	2	2	Rigid
**5WK3**	14.82	8.13	0.00	0.04	0	0	Rigid
**5WUX**	11.25	4.51	0.00	0.31	0	0	Rigid
**5X0T**	1.27	1.32	0.94	0.89	2	2	Rigid
**5Y9J**	7.09	2.76	0.07	0.42	0	1	Rigid
**6A77**	1.99	1.90	0.61	0.66	2	2	Rigid
**6B0S**	1.65	1.55	0.80	0.84	2	2	Rigid
**6BPC**	4.08	3.67	0.09	0.14	0	1	Rigid
**6CWG**	2.98	1.74	0.50	0.73	1	2	Rigid
**6DBG**	9.28	2.90	0.00	0.32	0	1	Rigid
**6OC3**	13.41	4.32	0.04	0.17	0	0	Rigid
**7CEI**	1.26	1.33	0.85	0.93	2	2	Rigid

References.

References

1. Vreven T, Moal IH, Vangone A, Pierce BG, Kastritis PL, Torchala M, Chaleil R, Jiménez-García B, Bates PA, Fernandez-Recio J, et al., Updates to the integrated protein-protein interaction benchmarks: Docking benchmark version 5 and affinity benchmark version 2. *Journal of Molecular Biology* **427**, 3031–3041 (2015).
2. Basu S, Wallner B, DockQ: A quality measure for protein-protein docking models. *PLOS ONE* **11**, 1–9 (2016).
